# Dental treatment needs in the Canadian population: analysis of a nationwide cross-sectional survey

**DOI:** 10.1186/1472-6831-12-46

**Published:** 2012-10-27

**Authors:** Chantel Ramraj, Amir Azarpazhooh, Laura Dempster, Vahid Ravaghi, Carlos Quiñonez

**Affiliations:** 1 Discipline of Dental Public Health, Faculty of Dentistry, University of Toronto, Toronto, ON, Canada; 2 Oral Health & Society Research Unit, Faculty of Dentistry, McGill University, Montreal, QC, Canada

**Keywords:** Dental care needs, Health policy, Socio-demographic/economic factors

## Abstract

**Background:**

Nationally representative clinical data on the oral health needs of Canadians has not been available since the 1970s. The purpose of this study was to determine the normative treatment needs of a nationally representative sample of Canadians and describe how these needs were distributed.

**Methods:**

A secondary analysis of data collected through the Canadian Health Measures Survey (CHMS) was undertaken. Sampling and bootstrap weights were applied to make the data nationally representative. Descriptive frequencies were used to examine the sample characteristics and to examine the treatment type(s) needed by the population. Bivariate logistic regressions were used to see if any characteristics were predictive of having an unmet dental treatment need, and of having specific treatment needs. Lastly, multivariate logistic regression was used to identify the strongest predictors of having an unmet dental treatment need.

**Results:**

Most of the population had no treatment needs and of the 34.2% who did, most needed restorative (20.4%) and preventive (13.7%) care. The strongest predictors of need were having poor oral health, reporting a self-perceived need for treatment and visiting the dentist infrequently.

**Conclusions:**

It is estimated that roughly 12 million Canadians have at least one unmet dental treatment need. Policymakers now have information by which to assess if programs match the dental treatment needs of Canadians and of particular subgroups experiencing excess risk.

## Background

Prior to the 2007/09 Canadian Health Measures Survey (CHMS), there was no nationally representative clinical data on the oral health needs of Canadians since the 1970/72 Nutrition Canada National Survey [1]. This is of concern as identifying the needs of a population is the primary step in the development and planning of programs [2]. In Canada, dental services are predominantly delivered in the private sector on a fee-for-service basis. Canadians are largely responsible for financing their own dental care, so enabling resources for obtaining care, such as income and insurance, dictate the use of dental services instead of the need for treatment. In this regard, it is especially important to identify the subgroups that have the greatest amount of unmet needs, in order to determine priorities for the most effective use of public resources. Without a clear picture of the burden of oral disease and how it is distributed, the danger of a top-down approach to providing health services arises, which relies heavily on what a few people perceive to be the needs of the population rather than what they actually are [3]. In short, this information is important as it can help policymakers understand the distribution of population needs, allowing them to compare current approaches to dental care with actual treatment needs.

Previous Canadian studies examining treatment needs have primarily focused on children and the elderly [4-6]. Generally, the oral health status of at risk children and adolescents appears to be poor resulting in the need for several treatments including urgent, restorative, periodontal and preventive care [4-6]. Similarly, very high levels of unmet need among older adults have been observed regardless of whether they live in an institution or are homebound [7]. The treatments they require have been reported as consisting of urgent, preventive, periodontal, restorative and prosthodontic care [7]. Other studies of need have also relied on self-reported information. Although patient self-reports are the most convenient mechanism for obtaining first-hand health information, they have been found to be heavily influenced by personal beliefs, cultural background, and social, educational, and environmental factors [8]. Furthermore, regarding treatment needs, self-reports have been found to often provide different assessments from those of clinically determined standards [8]. For example, it has been found that people are usually unable to report signs and symptoms related to periodontal conditions [8,9].

This paper outlines the normative dental treatment needs of the Canadian population. It examines if any characteristics are predictive of having unmet dental treatment needs, and the type of treatment needed. It applies a modified version of Andersen’s emerging model of health services utilization to determine which factors are the strongest predictors of having an unmet dental treatment needs. The goal is to inform policymakers of the levels and distribution of treatment needs in the Canadian population.

## Methods

### Study design and sample

This study was a secondary data analysis of the 2007/09 Canadian Health Measures Survey (CHMS), Cycle 1 Household and Clinic Questionnaires, which was a cross-sectional survey. Data were accessed from Statistics Canada’s Research Data Centre (RDC) at the University of Toronto. The CHMS collected health measures from approximately 5,600 people, which statistically represented 97% of the Canadian population between 6 and 79 years of age. This consisted of those living in privately occupied dwellings in Canada’s ten provinces and three territories. Those excluded from the survey included persons living on Indian Reserves or Crown lands, residents of institutions, full-time members of the Canadian Forces and residents of certain remote regions [1]. Health Canada’s Research Ethics Board, the Office of the Privacy Commissioner of Canada, and the Data Access and Control Services Division at Statistics Canada provided ethical review and consulted the CHMS on ethical, social and legal issues. The details regarding these issues are outlined by Day, Langlois, Tremblay & Knoppers (2007) [10]. The CHMS employed four forms: a consent form for respondents aged 20 to 79 years, for respondents aged 14 to 19 years and for parents/guardians of respondents aged 6 to 13 years, and an assent form for respondents aged 6 to 13 years [10].

### Collection of data

Data collection was conducted by Statistics Canada, in partnership with Health Canada and the Public Health Agency of Canada, between March 2007 and February 2009. A personal interview using a computer-assisted interviewing method was employed, followed by a visit to a mobile examination centre for direct clinical measurement of various health outcomes, including oral health. The country was divided into 257 potential collection sites. A collection site was defined as a “geographic area with a population of >10,000 where each potential respondent had a maximum travel distance to the clinic of 100 km or less” [1]. Within each site, “dwellings with known household composition (from the 2006 census) were divided into 6 strata to obtain sufficient numbers of people in each of the targeted age groups and a random sample of dwellings from each stratum was taken” [1].

For the household interview, the interviewer randomly selected one or two respondents and conduced an interview lasting about 45 to 60 minutes. Thirty-four specific oral health questions were asked that gathered data related to oral health, such as oral symptoms, dental care habits, and source of funds to pay for dental care. Additionally, relevant sections of the interview gathered information on socio-demographic information [1].

The Department of National Defence supplied 12 dentist-examiners for the two-year collection period who were calibrated to World Health Organization (WHO) standards by a gold standard trainer [1]. Inspections of all clinic staff and on all components of the examination were performed at regular intervals to provide a direct assessment of protocol adherence, communication with participants, overall data collection quality and operation of the clinic [1]. Prior to the start of the oral assessment, the examining dentist asked the participant 18 questions regarding dental symptoms (pain, bleeding, dry mouth, etc.) [1]. Additionally, 15 medical history questions were asked to ensure that the participant could undergo the clinical evaluation and those with acute or chronic conditions were not examined [1]. The treatment needs of the participant was also assessed and ranked according to urgency [1]. Specific groupings were used in order to classify each type of treatment need (see Table 1). Overall, the oral health assessment was completed on 5,586 people [1].

**Table 1:** Groupings used to assess each treatment type

**Description**	**Examples**
Preventive	Examination; prophylaxis; fluoride; sealant; radiographs
Restorative	Fillings; crowns; bridges for restoration of carious lesions
Periodontic	Scaling; root planning; periodontal surgery
Endodontic	Root canal therapy
Prosthodontic	Removable/fixed, partial/full dentures; implant, bridge or crown
Orthodontic	Under treatment, requiring orthodontic care as defined
Other	Something of significance not otherwise able to be coded; TMD, esthetics and soft tissue (added for this study)
Urgent	Treatment needed within a week

**Note:** Adapted from The Oral Health Needs Assessment took kit provided by Health Canada: http://www.fptdwg.ca/ohnat/index.php.

### Data variables and analysis

All of the variables used in this study were imported into SPSS (Statistical Package for the Social Sciences) for Windows (release 18.0, IBM Corporation, Armonk, NY) from the original CHMS Wave 1 master data file. The SPSS data file containing all of the variables of interest was then imported into STATA for Windows (release 12.0, StataCorp LP 2012) for data analysis. All cases where participants were not clinically examined (N=18) were excluded from the analysis. As specified by Statistics Canada, all analysis that produced small cell sizes (<10) could not be released from the RDC.

In order for the data to be representative of the population, a unique survey weight was assigned to each participant that corresponded to the number of people represented by that participant in the population as a whole. To account for the complex sampling design, bootstrap weights were also applied to obtain reliable estimates and variances representative of Canada. A total of 500 bootstrap weights were applied since the sample was allocated over 10 age-sex groups, and it was estimated that 500 units per group was required to produce national estimates, for a total of 5,000 reporting units.

**Figure 1:**
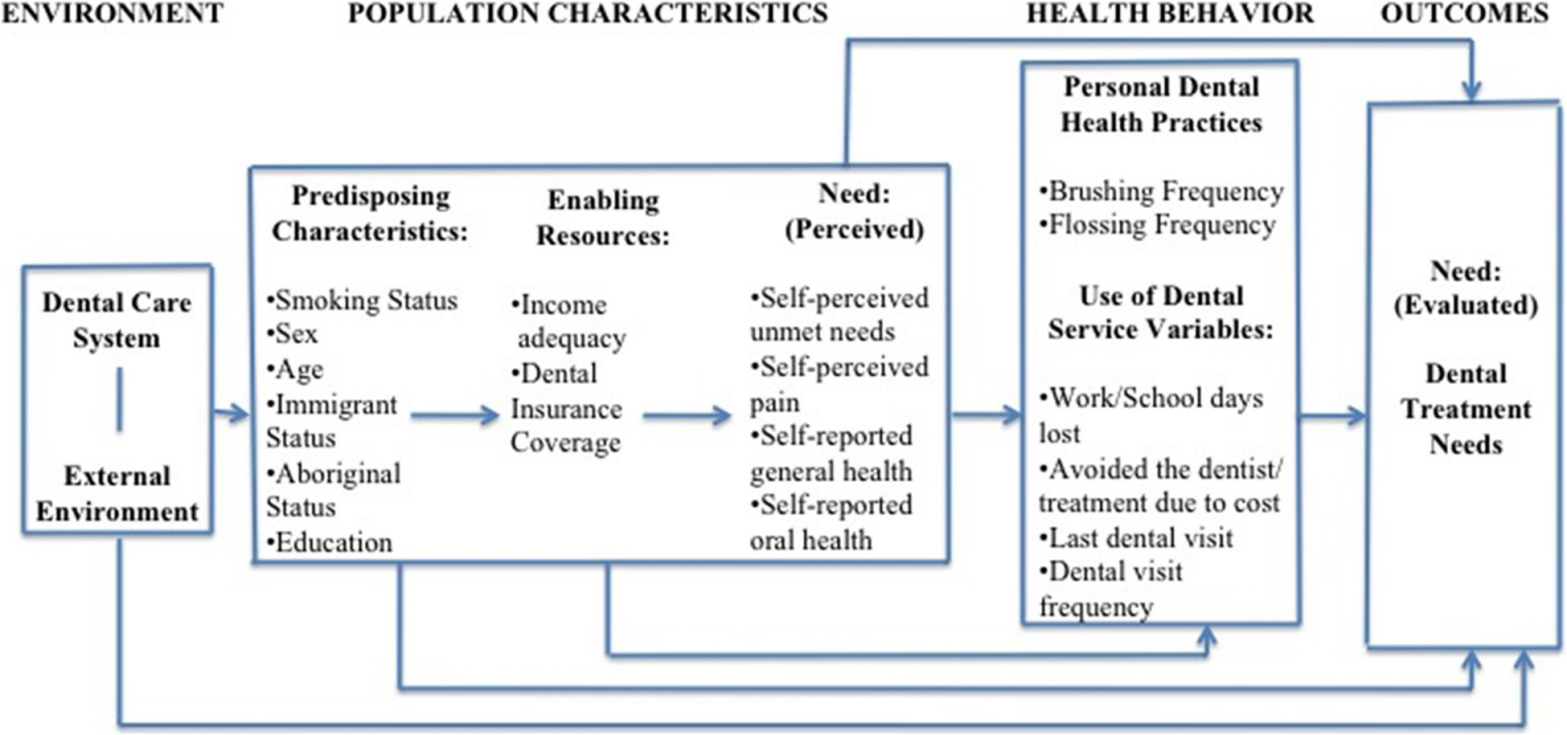
Operational model - Modification of Andersen’s Emerging Model (adapted from Andersen, 1995).

The dependent variable in this study was the clinically examined dental treatment need(s) of the participant. In some of the analysis this outcome variable was dichotomized into ‘yes’ (if the participant had at least one treatment need) and ‘no’ (if the participant had no treatment needs). In other sections of the analysis, the outcome variable was displayed by type of treatment (prevention, fillings, surgery, periodontics, endodontics, prosthodontics, orthodontics, other and urgent needs). The independent variables selected for this study were based on a modified version of Andersen’s emerging model of health services utilization. Figure 1 shows how this model was used to arrange variables under five headings: Predisposing, enabling, need, personal dental health practices, and use of dental services. Income adequacy was based on the total household income and the number of people living in the household. Table 2 shows the criteria used by the CHMS to differentiate between each income category. For insurance, participants who had an employer-sponsored plan or a private dental plan were grouped to form the ‘privately insured’ group. Those included under ‘publically insured’ were covered under a provincial program (for children or seniors), or a government program for social service (welfare) clients or First Nations and Inuit people. Those who had no public/private coverage and paid for dental care out-of-pocket were ‘non-insured.’

**Table 2:** Income adequacy categories defined

**Description**	**Household Size**	**Household Income**
Lowest income grouping	1 or 2 people	$0-$14,999
	3 or 4 people	$0-$19,999
	>4 people	$0-$29,999
Middle income grouping	1 or 2 people	$15,999-$59,999
	3 or 4 people	$20,000-$79,999
	>4 people	$30,000-$79,999
Highest income grouping	1 or 2 people	$60,000-$100,000+
	>2 people	$80,000-$100,000+

Descriptive frequencies were used to examine the sample characteristics and to examine the treatment type (prevention, restorations, surgery, periodontics, endodontics, prosthodontics, orthodontics, other and urgent) needed by the population.

Bivariate analyses were used to examine if any characteristics (predisposing, enabling, need etc.) were predictive of having any unmet dental treatment needs, and of having a specific treatment need (prevention, restorations, surgery, etc.). These were bivariate regressions and therefore did not adjust for other factors.

Multivariate logistic regressions were employed to determine which factors (predisposing, enabling, need, etc.), were the strongest predictors of having at least one unmet need. Using the modified Andersen model [11], five models were used to compute odds ratios for having an unmet need. These models progressively adjusted for predisposing, enabling, need, personal dental health practice, and use of dental service factors, as done by Al Snih et al., (2006) [12]. Prior to being entered into the model, each independent variable was tested with all of the other independent variables to check for any possible correlation amongst and between the predictor variables. The variance inflation factor (VIF), which quantifies the severity of multicollinearity, was found to be low (<3) for each variable and all of variables were found to be insignificant (p<0.25) at the bivariate level. Therefore, every variable outlined in the modified Andersen model was entered simultaneously as blocks into the multivariate logistic regressions. Model 1 included only the predisposing factors. Model 2 added the enabling factors with all of the previously entered predisposing factors. Similarly, Models 3, 4 and 5 added in the perceived need variables, the personal dental health practices, and the use of dental service variables, respectively.

## Results

The final sample included 5,586 participants, representing 29,157,460 Canadians when weighted, out of a current population of 33,476,688 [13]. Table 3 shows that the sample consisted of an approximately even number of males (49.9%) and females (50.1%), the majority of the participants were 20 to 39 (30.9%) and 40 to 59 (33.5%) years of age, and slightly over half had never smoked (52.6%). Roughly an equal proportion of the population had a degree or diploma (49.6%), the majority was born in Canada (79.0%) and were non-Aboriginal (96.9%). Most were in the highest income category (47.9%) and had private dental insurance coverage (62.3%). The majority reported their oral health and general health as excellent to good (respectively, 84.5% and 91.9%), rarely or never experienced oral pain (88.4%), and most did not perceive a need for dental treatment (67.0%). The majority said that they had visited the dentist in the last year (74.5%), and that they had not avoided recommended dental treatment in the past year due to cost (83.5%).

**Table 3:** Sample characteristics of Canadian population 2007–2009 (N=29,157,460)

**N= 29,157,460**	**%**
**Sex**	
Male	49.9
Female	50.1
**Age**	
6 to 11	7.4
12 to 19	11.4
20 to 39	30.9
40 to 59	33.5
60 to 79	16.8
**Smoking Status**	
Never smoked	52.6
Past smoker	27.1
Current smoker	20.3
**Education**	
Degree/diploma	49.6
<Degree/diploma	50.5
**Immigrant Status**	
Born in Canada	79.0
Not born in Canada	21.0
**Aboriginal Status**	
Non-Aboriginal	96.9
Aboriginal	3.10
**Income adequacy**	
Highest income	47.9
Middle income	31.9
Lowest income	20.3
**Dental insurance**	
Private coverage	62.3
Public coverage	5.8
Non-insured	31.9
**Self-reported oral health**	
Excellent/very good/good	84.5
Fair/poor	15.5
**Self-reported general health**	
Excellent/very good/good	91.9
Fair/poor	8.1
**Self-reported oral pain**	
Rarely/never	88.4
Often/sometimes	11.6
**Self-perceived unmet needs**	
No needs	67.0
Has at least one need	33.0
**Brushing frequency**	
>Once/day	72.0
Once/day	24.6
<Once/day or Never	3.4
**Flossing Frequency**	
>Once/day	8.6
Once/day	21.4
<Once/day	42.0
Never	28.1
**Avoided dental treatment due to cost**	
No	83.5
Yes	16.5
**Last Dental Visit**	
In the last year	74.5
More than one year ago	25.5
**Dental Visit Frequency**	
> Once a year	42.6
Once a year	31.7
< Once a year	9.2
Only for emergency	13.3
Never	3.2
**Work/School Days Lost**	
No	60.9
Yes	39.2

**Figure 2:**
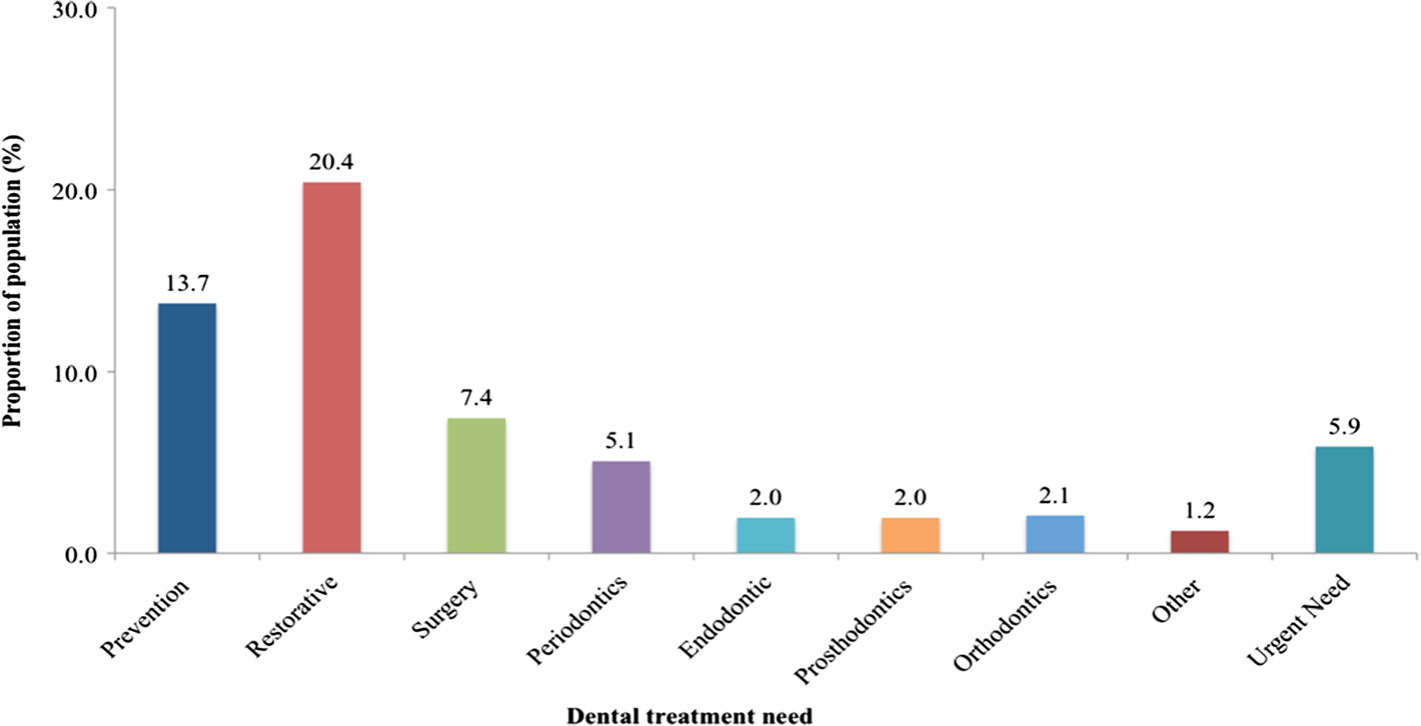
Percent of type of dental treatment required in the Canadian population.

Most of the sample had no treatment needs (65.8%). Of the 34.2% who did require treatment, 19.4% were found to have one dental treatment need and 14.6% required more than one need, representing close to 5 million people. Looking into the types of dental treatments needed, Figure 2 displays that most needed restorative (20.4%) and preventive (13.7%) care. Approximately 6.0%, representing nearly 2 million people, had an urgent need (i.e. treatment was required within one week).

As displayed in Table 4, all of the variables, with the exception of immigrant status, were significant predictors of having an unmet dental treatment need. Those reporting their oral health as fair or poor were 5.9 times more likely to have an unmet need than those reporting excellent or good oral health (95% CI=4.3-8.0, P=0.001). Those who perceived a need for treatment were 4.6 times more likely to have a treatment need than those who did not (95% CI=3.7-5.8, P=0.001).

**Table 4:** Percent and unadjusted odds ratios of individuals who have at least one clinically determined treatment need by each independent factor

	**%**	**Unadjusted OR (95% CI)**	**P-value**
**Predisposing Factors**			
**Sex**			
Male (Reference)	36.3		
Female	29.3	0.7 (0.6, 0.9)	0.002
**Age**			
6 to 11 (Reference)	26.3		
12 to 19	28.6	1.1 (0.8, 1.5)	0.398
20 to 39	34.5	1.5 (1.2, 1.8)	0.002
40 to 59	35.1	1.5 (1.3, 1.8)	0.001
60 to 79	30.7	1.3 (1.0, 1.5)	0.030
**Smoking Status**			
Never smoked (Reference)	29.4		
Past smoker	31.2	1.1 (0.8, 1.5)	0.590
Current smoker	46.3	2.1 (1.6, 2.6)	0.001
**Education**			
Degree/Diploma (Reference)	28.9		
<Degree/Diploma	36.5	1.4 (1.1, 1.9)	0.016
**Immigrant Status**			
Born in Canada (Reference)	31.8		
Not born in Canada	36.6	1.2 (0.9, 1.7)	0.174
**Aboriginal Status**			
Non-Aboriginal (Reference)	32.4		
Aboriginal	46.6	1.8 (1.0. 3.3)	0.044
**Enabling Factors**			
**Income adequacy**			
Highest income (Reference)	26.1		
Middle income	35.7	1.6 (1.3, 1.9)	0.001
Lowest income	43.0	2.1 (1.6, 2.9)	0.001
**Dental insurance**			
Private coverage (Reference)	27.2		
Public coverage	47.6	2.4 (1.6, 3.6)	0.001
Non-insured	41.2	1.9 (1.5, 2.3)	0.001
**Need Factors**			
**Self-reported oral health**			
Excellent/very good/good (Reference)	26.4		
Fair/poor	67.8	5.9 (4.3, 8.0)	0.001
**Self-reported general health**			
Excellent/very good/good (Reference)	31.4		
Fair/poor	45.0	1.8 (1.3, 2.6)	0.005
**Self-reported oral pain**			
Rarely/Never (Reference)	31.4		
Often/Sometimes	43.7	1.7 (1.3, 2.2)	0.001
**Self-perceived unmet needs**			
No Needs (Reference)	21.5		
Has at least one need	55.8	4.6 (3.7, 5.8)	0.001
**Personal Dental Health Practices**			
**Brushing frequency**			
>Once/day (Reference)	28.9		
Once/day	42.0	1.8 (1.5, 2.1)	0.001
<Once/day or Never	50.0	2.5 (1.7, 3.6)	0.001
**Flossing Frequency**			
>Once/day (Reference)	22.4		
Once/day	29.1	1.4 (0.9, 2.2)	0.102
<Once/day	29.2	1.4 (0.9, 2.4)	0.153
Never	44.2	2.8 (1.7, 4.4)	0.001
**Use of Services**			
**Avoided dental treatment due to cost**			
No (Reference)	29.0		
Yes	52.5	2.7 (2.2, 3.3)	0.001
**Last Dental Visit**			
In the last year (Reference)	26.0		
More than one year ago	51.3	3.0 (2.3, 3.9)	0.001
**Dental Visit Frequency**			
>/=Once a year (Reference)	26.3	3.0 (2.4, 3.8)	0.001
<Once a year/emergency/never	51.8		
**Work/School Days Lost**			
No (Reference)	36.5		
Yes	27.1	0.7 (0.5, 0.8)	0.001

According to Table 4, those who reported poor oral health, a perceived need for treatment, and had poor dental visiting habits, were found to have the highest odds of requiring dental treatment. This finding was also present for each treatment type (data not shown). Table 5 shows that those reporting their oral health as fair or poor, and those that perceived a need for dental treatment, were 4.6 (95% CI=3.7-5.7, P=0.001) and 4.5 (95% CI=3.4-6.1, P=0.001) times more likely than their counterparts, respectively, to require restorative treatment, the most prevalent needed treatment found in the sample. Also, those who saw a dental professional more than one year ago (OR=3.0, 95% CI=2.3-3.8, P=0.001) and tended to visit the dentist less than once a year or only for emergencies or never (OR=3.0, 95% CI=2.3-3.8, P=0.001), were both more likely than their counterparts to need restorative care.

**Table 5:** Percent and unadjusted odds ratio of individuals who have restorative needs

	**%**	**Unadjusted OR (95% CI)**	**P-value**
**Self-reported oral health**			
Excellent/good (Reference)	15.7		
Fair/Poor	46.0	4.6 (3.7, 5.7)	0.001
**Self-perceived unmet needs**			
No Needs (Reference)	11.8		
Has at least one need	37.8	4.5 (3.4, 6.1)	0.001
**Last Dental Visit**			
In the last year (Reference)	15.1		
More than one year ago	34.7	3.0 (2.3, 3.8)	0.001
**Dental Visit Frequency**			
>/=Once a year (Reference)	15.3		
<Once a year/emergency/never	34.9	3.0 (2.3, 3.8)	0.001

Finally, to determine the strongest predictors of dental treatment need, five models were used to progressively adjust for the factors in the modified Andersen model (predisposing, enabling, need, personal dental health practice and use of dental service factors). Table 6 shows the results obtained from the last and final model (Model 5), which entered all of the factors. After adjustment, being male, a current smoker, having less than a degree or diploma, having public insurance coverage, never flossing, and reporting a last dental visit of more than one year ago, were significant predictors of having an unmet dental treatment need. The strongest predictors were need variables. Those who reported their oral health as fair or poor and had a self-perceived need for treatment were nearly three (OR=2.9, 95% CI=1.8-4.6, P=0.001) and three-and-a-half (OR=3.4, 95% CI=2.3-4.9, P=0.001) times more likely to have an unmet need.

**Table 6:** Multivariate logistic regressions predicting the odds of having at least one clinical need including predisposing, enabling, need, personal dental health practice and use of service factors (N=23,456,538)

	**Model 5 (N=23,456,538)**	
	**OR (95% CI)**	**P-value**
**Predisposing Factors**		
**Sex**		
Male (Reference)		
Female	0.7 (0.6, 0.9)	0.004
**Age**		
12 to 19 (Reference)		
20 to 39	1.0 (0.7, 1.5)	0.841
40 to 59	1.3 (0.8, 2.1)	0.190
60 to 79	1.0 (0.7, 1.4)	0.841
**Smoking Status**		
Never smoked (Reference)		
Past smoker	1.0 (0.7, 1.5)	0.940
Current smoker	1.3 (1.0, 1.8)	0.040
**Education**		
Degree/Diploma (Reference)		
<Degree/Diploma	1.4 (1.1, 1.8)	0.026
**Immigrant Status**		
Born in Canada (Reference)		
Not born in Canada	1.2 (0.8, 1.7)	0.412
**Aboriginal Status**		
Non-Aboriginal (Reference)		
Aboriginal	1.4 (0.8, 2.5)	0.236
**Enabling Factors**		
**Income adequacy**		
Highest income (Reference)		
Middle income	1.2 (0.9, 1.5)	0.149
Lowest income	1.1 (0.8, 1.4)	0.630
**Dental insurance**		
Private coverage (Reference)		
Public coverage	2.0 (1.3, 3.1)	0.005
Non-insured	1.4 (1.1, 1.7)	0.023
**Need Factors**		
**Self-reported oral health**		
Excellent/Good (Reference)		
Fair/Poor	2.9 (1.8, 4.6)	0.000
**Self-reported general health**		
Excellent/Good (Reference)		
Fair/Poor	1.2 (0.8, 1.7)	0.324
**Self-reported oral pain**		
Rarely/Never (Reference)		
Often/Sometimes	0.8 (0.5, 1.1)	0.138
**Personal Dental Health Practices**		
**Brushing frequency**		
>Once/day (Reference)		
Once/day	1.2 (0.9, 1.7)	0.150
<Once/day or Never	0.8 (0.4, 1.7)	0.577
**Flossing Frequency**		
>Once/day (Reference)		
Once/day	2.0 (1.1, 3.6)	0.133
<Once/day	1.6 (0.9, 3.0)	0.108
Never	2.4 (1.3, 4.6)	0.011
**Use of Services**		
**Avoided dental treatment due to cost**		
No (Reference)		
Yes	1.2 (0.8, 1.8)	0.313
**Last Dental Visit**		
In the last year (Reference)		
More than one year ago	2.0 (1.4, 3.0)	0.002
**Dental Visit Frequency**		
>/=Once a year (Reference)		
<Once a year/emergency/never	1.2 (0.8, 1.8)	0.476
**Work/School Days Lost**		
No (Reference)		
Yes	1.1 (0.8, 1.4)	0.644

## Discussion

This study is the first in approximately 40 years to explore representative clinical information on the dental treatment needs of Canadians. Its findings suggest that policymakers should be concerned that an estimated 12 million Canadians had an unmet dental treatment need, that close to 5 million had numerous unmet needs, and that nearly 2 million had an urgent need (i.e. treatment was required within one week). With most people requiring restorative and preventive care, these findings reflect those of Quiñonez and Locker (2007) [14], who found that 26% of the Canadian adult population self-reported having a cost-prohibitive (i.e. unmet) dental need, with fillings and preventive procedures (cleanings and check-ups) being the most prevalent unaffordable needs.

Poor self-rated oral health, a perceived need for dental treatment, and infrequently visiting a dental professional were the main predictors of having unmet dental treatment needs. This seems counterintuitive since one would expect that those who are conscious of their poor oral health and requirement for treatment would visit the dentist in order to meet their needs. However, here we see the ‘paradox of need’, as noted by Muirhead et al. (2009) [15], who found that working poor Canadians who reported the worst oral health or who had a perceived need for treatment, were the lowest dental service users. Studies have shown that people with poor oral health and a perceived need for treatment may avoid dental visits because of the barriers imposed by the costs of dental treatment, the anxiety of potential pain, or concern about being judged by dentists for their poor oral condition [15,16]. Unfortunately, aside from avoiding dental treatment for cost reasons, which was found to be the case for around 17% of the sample population, the CHMS did not collect information regarding any other potential reasons for not visiting the dentist (e.g. anxiety, fear of judgement, etc.).

This study also found that those who had access to public programs were worse off in terms of having unmet dental needs, when compared to those with private insurance or without any insurance coverage. The National Survey of Adult Oral health 2004–06 conducted in Australia found something similar, noting that untreated decay was more frequent among those eligible for public dental care [17]. These findings support a key issue noted by Leake and Birch (2008) [18], who stated that although the public funding of dental care provides a means of overcoming the divergence between the ability to pay for care and need for care, having such coverage is not enough. There are other factors at play other than just affordability, such as the availability, accommodation, and acceptability of dental care. For example, as noted by Quiñonez et al., (2010) [19], Canadian dentists have consistently voiced their dissatisfaction with public insurance plans, and approximately a third have reported limiting the number of patients they accept who have public insurance. Therefore, solely increasing public subsidies for these groups may not fully increase their utilization of dental services unless other access issues are also addressed.

It is important to consider the limitations of this study. Firstly, it is difficult to know the true extent of treatment need in the Canadian population considering the specific details of need were not collected by the CHMS. For example, multiple treatment needs of the same type were recorded as a single need (e.g. a participant that required a single restoration was reported similarly to a participant with three restorations). Also, this study cannot support conclusions about the causal effects of any predisposing, enabling, need, personal dental health, and use of dental service factors, since it is based on a cross-sectional survey. Grouping children and adults together in the analysis is recognized as a limitation, as there are potential differences in treatment needs. The variable used for last dental visit was dichotomized into two categories, and the cut-off point of visited “in the last year” is noted to be a limitation of the study. Information on those who visit the dentist once every two, three years, etc., could have been explored to observe whether there would be changes in the severity of treatment need. Lastly, it is important to recall that certain populations who are known to have high levels of dental disease and limited access to dental care (e.g. Aboriginal populations and seniors in institutions) were not included in the CHMS. Therefore, the 34% of the population found to require dental treatment is most likely an underestimation of the true need present in the Canadian population.

## Conclusion

This study highlights the importance of an ongoing surveillance system to measure the oral health status of populations, oral health inequalities, and other trends. Internationally, it still remains a challenge for most countries to establish a database for the clinical monitoring and surveillance of oral health [20]. In order to predict the future burden of oral disease, as well to identify potential interventions to reduce the burden of these diseases, data collection and reporting standards are needed to ensure that data is consistently collected and used effectively to inform policy, prevention and control activities for health [20]. Without this information, the capacity to develop oral health policy based on evidence is limited. Therefore, the WHO recommends that regular clinical oral health surveys be conducted every 5–6 years in the same community or setting [20]. In this regard, the current study provides valuable baseline information on the dental treatment needs of most Canadians, and highlights the greatest areas of unmet need. This knowledge can now be used by program and policymakers to develop or refine targeted dental health programs in an effort to improve the oral health of the Canadian population as a whole.

## Abbreviations

CHMS: Canadian Health Measures Survey; RDC: Research Data Centre; WHO: World Health Organization; SPSS: Statistical Package for the Social Sciences; VIF: Variance Inflation Factor.

## Competing interests

The authors declare that they have no competing interests.

## Authors’ contributions

CR, CQ, LD and AA contributed to the conception and design of the study. CR was granted permission to access the RDC in order to analyze and interpret the data, and drafted the manuscript. VR provided statistical assistance and helped with the interpretation of data. CQ revised the manuscript critically for important intellectual content. All authors read and approved the final manuscript.

## Pre-publication history

The pre-publication history for this paper can be accessed here:

http://www.biomedcentral.com/1472-6831/12/46/prepub
